# War, displacement, and the best location for temporary sheltering: a qualitative study

**DOI:** 10.1186/s12889-022-14495-w

**Published:** 2022-11-11

**Authors:** Reza Ramazani, Arezoo Yari, Ahad Heydari, Ahmad Ali Hanafi-Bojd, Ahmad Soltani, Shahbakhti Rostami, Abbas Ostadtaghizadeh

**Affiliations:** 1 Department of Health in Emergencies and Disasters, School of Public Health, Tehran University of Medical Sciences, Tehran, Iran; 2 Social Determinants of Health Research Center, Research Institute for Health Development, Kurdistan University of Medical Sciences, Sanandaj, Iran; 3 Department of Health in Emergencies and Disasters, School of Medicine, Kurdistan University of Medical Sciences, Sanandaj, Iran; 4 Department of Medical Entomology and Vector Control, School of Public Health, Tehran University of Medical Sciences, Tehran, Iran; 5 Zoonoses Research Center, Tehran University of Medical Sciences, Tehran, Iran; 6 Iran-Helal Institute of Applied-Science and Technology, Red Crescent Society of the Islamic Republic of Iran, Tehran, Iran; 7 Research Center for Health Management in Mass Gathering, Red Crescent Society of the Islamic Republic of Iran, Tehran, Iran; 8 Karaj Payam Noor University, Islamic Republic Of, Karaj, Iran; 9 Poorsina Ave, Public Health Faculty, Health in Disaster and Emergency Department, Tehran, 14177-43578 Iran

**Keywords:** Conflict, Displacement, Refugees, Sheltering; Iran

## Abstract

**Background:**

One of the most important consequences of man-made disasters in the world is the loss of homes and, ultimately, forced population displacement. The sheltering of forcibly refugees to safe areas requires the study of scientific criteria.

**Methods:**

This qualitative content analysis study was conducted in Iran to identify the criteria for the sheltering of refugees due to conflict. Participants in the study comprised experts and managers who had expertise, experience, or knowledge in the shelter for refugees. Data was collected in semi-structured interviews with 20 individuals and analyzed by qualitative content analysis to extract and categorize the factors affecting sheltering for refugees.

**Results:**

The results of this study showed that a wide range of factors should be considered in the sheltering of refugees due to conflict, including land type, access to facilities, access to infrastructures, homogeneity, and similarities, security, distance from danger, environmental factors, economic issues, and political and legal issues. These nine categories covered 53 subcategories and 188 factors.

**Conclusions:**

The comprehensiveness of the factors mentioned in this study showed that the sheltering of refugees due to conflict requires planning, training, culture building, promoting readiness, and inter-organizational coordination. Moreover, managers, policymakers, decision-makers in relief agencies, humanitarian organizations, and organizations involved in sheltering of refugees due to conflict should attend to these factors to improve the process and prevent problems.

## Background

Experiences and trends in forced population displacement due to disasters in the world show that a large number of people are losing their homes every year. A report by the United Nations High Commissioner for Refugees (UNHCR) indicates that the number of people forcibly displaced due to war, conflicts, and public order offenses reached 79.5 million by the end of 2019. This upward trend shows that the number of forcibly displaced persons has almost doubled in the decade from 2010–2019, which is named the Decade of Displacement. Examples of such forced population displacements in which people have lost their homes and main shelters in life include the crises in the Syrian conflict, South Sudan, Ukraine, Myanmar refugees in Bangladesh, and renewed conflict and security concerns in Afghanistan, Iraq, Libya, Somalia, and the great humanitarian crisis and forced displacement of persons in Yemen [1]. Iran has a history of hosting over one million displaced persons from Afghanistan and Iraq [2]. Many countries, including Iran, comply with international requirements and commitments to accept foreign refugees and internally displaced persons (IDP) according to humanitarian principles and laws [3].

Wars and natural disasters are currently some of the main factors threatening human life. Therefore, key elements in dealing with these disasters are safety and preparedness [4]. It is necessary for governments, humanitarian agencies, domestic organizations, and people and associations to be prepared for a positive response to crises or situations of insurrections and wars, and reduce any potential future risks [5]. This is in line with the objectives of the Sendai Framework for Disaster Risk Reduction 2015–2030 (Sendai Framework) which commits Member States to prevent and reduce the risk of disasters [6]. Considering that during the conflicts, large people lose their home and are forced to leave their place of residence and migrate to other regions or other countries [1]. Providing suitable shelter for refugees is central to reducing vulnerability and promoting community resilience during crises. This is at the core of humanitarian activities. According to humanitarian charters, everyone has the right to adequate shelter [5]. In addition, temporary shelter is essential for security, well-being, protection against metrological hazard, health protection, and disease prevention [7]. By providing proper and basic shelter can help them to maintain their health and protect their lives and reduce the spread of communicable and vector-borne diseases among refugees and also host communities. In other words, providing shelter, water, sanitation, nutrition and psychological care, has important role in saving and preserving health and human life [5, 8]. In other words, shelters play an important role in reducing the health effects of disasters, and the lack of shelter or inadequate shelter leads to an increase in human casualties due to crises [7]. To respond to the maximum needs of refugees, a suitable site [9] and a plan to accommodate them are required in order to reduce their pain and suffering [10, 11]. Selecting a suitable site to accommodate refugees plays a key role in reducing the dangers to which they are exposed. A lack of suitable criteria can in fact create unpredictable situations, negatively affecting relief and support response for refugees [12].

Although some studies have examined the accommodation and sheltering of vulnerable persons affected by natural disasters which may even bear similarities to the accommodation criteria of refugees in manmade conflicts, few studies have specifically examined the shelter site selection criteria for refugees displaced by conflict. One such study is the Jana Abikova 2020 research conducted in the fuzzy method to examine the criteria set for refugee camps. The study introduced five categories (geographical, infrastructural, danger-related, social, and operational) and 20 subcategories for refugee camps [13]. Another study on the subject is that of Drakaki et al. conducted in 2018 in which three main categories (land type, support factors and position) and 11 subcategories were introduced [14]. The last of such studies found is the one conducted by Çetinkaya et al. in 2016. The criteria for the sheltering of persons displaced due to conflict were introduced in four main categories (geographical, social, infrastructural, and danger-related), and 19 subcategories based on the geographic information system [15]. Identifying all the variables and factors related to a phenomenon requires a comprehensive understanding of the phenomenon [16].

To the best of the researchers’ knowledge, review of literature did not yield any studies on the criteria for the sheltering of refugees due to conflict except some limited research. Therefore, in order to get a broader perspective of the criteria and properties of temporary shelters for displaced people due to war, in this qualitative study, using in-depth interviews, we tried to use experts' and officials’ opinions. The people interviewed were based in and residents of the border areas of Iran where have been subjected to the arrival of refugees in the past. Due to several reasons, Iran can be a good place to conduct this study. Among these reasons, the following can be mentioned: The strategic and geographical location of Iran in the Middle East region [16], the existence of war and conflict along Iran's borders [17], the history of the arrival of a large population of Afghan and Iraqi refugees from the western and eastern borders into the country in the past [2]. The results of this research can be used in creating and design of temporary shelters for refugees. The aim of the study is to set the criteria for the temporary sheltering of refugees due to conflict from the perspective of experts and opinion holders with a qualitative content analysis method.

## Methods

As it is necessary to have a comprehensive understanding of the criteria for the sheltering of refugees due to conflict in Iran, the present qualitative research was conducted in 2020–2021 with the aim of setting the criteria for the temporary sheltering of refugees due to conflict. Qualitative studies are suitable for identifying the variables pertaining to a phenomenon [15]. In this study, the views of experts and opinion holders on the subject were used. After the purposive sampling of participants, interviews were conducted, recorded, transcribed, and coded by the research team. Data was analyzed to extract and categorize the criteria for the temporary sheltering of refugees due to conflict.

### Participants

**Fig. 1:**
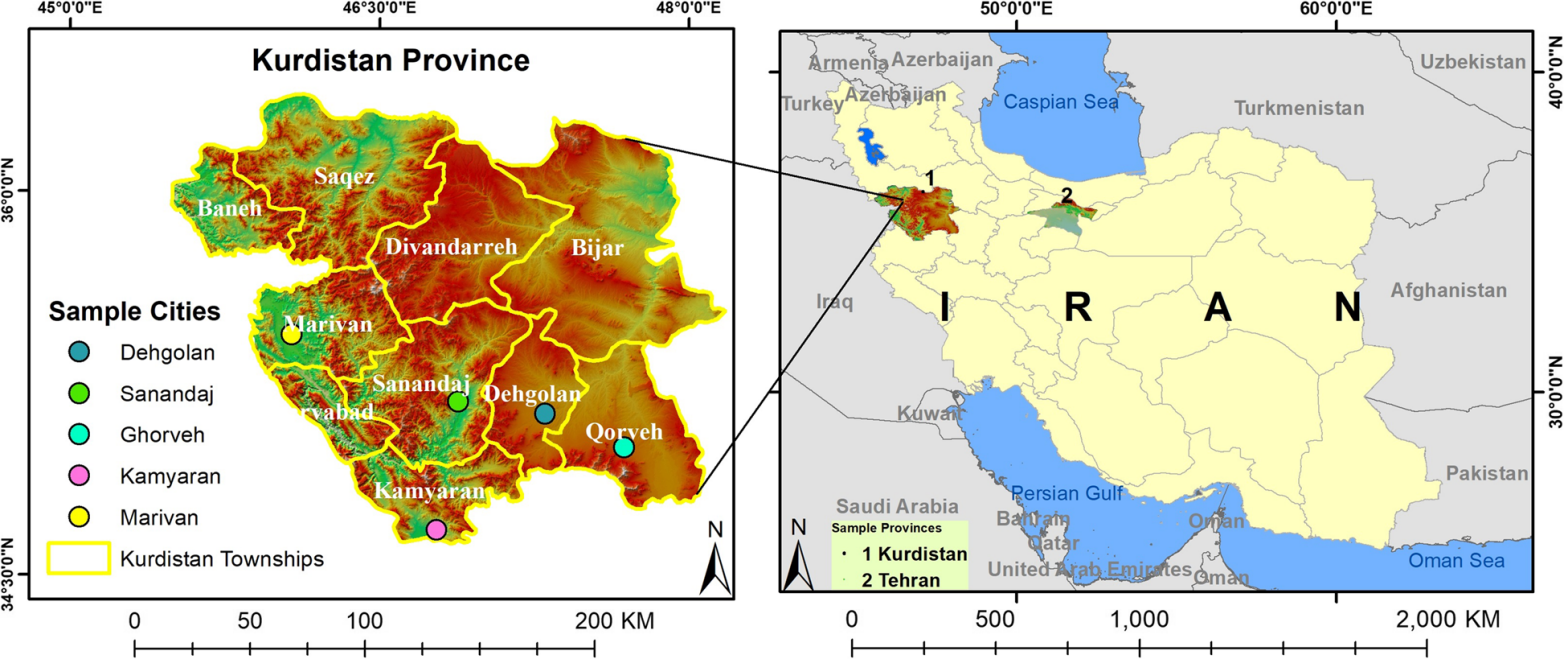
This map shows Iran's administrative borders and Iran's political borders with neighboring countries. The marked regions represent Tehran province, Kurdistan, and the cities of Kurdistan Province, where the research took place. Also, this map was constructed by the authors

From the start of the present study, a range of state organizations and departments involved in sheltering refugees due to conflict were identified, including the Ministry of Health and Medical Education, Iranian Red Crescent Society, Bureau for Aliens and Foreign Immigrants’ Affairs, Governor's Office, Water and Wastewater Company, Power Generation and Distribution Company, Ministry of Agricultural Jihad, and scientific research centers in Tehran, Karaj, and the border and central cities of Kurdistan Province in Iran (Fig. 1). The participants were then selected purposively and interviewed from among experts and managers in the mentioned organizations who possessed the knowledge, expertise, and experience in sheltering refugees due to conflict and were interested in taking part in the study. A main reason for selecting these people for the interview was the relevance of their organizations' services for refugees, as well as the effectiveness of their decision in sheltering. Another reason was emphasis of the High Commissioner for Refugees in UN on using the opinions of organizations of host countries and local communities in site selection [5, 11]. Their main knowledge, expertise, and experience included healthcare, relief work, political-security response, health expertise in accidents and disasters, immigrant affairs, and service companies for water, electricity, and agriculture. we also tried to use the opinions of academics because these people had valuable opinions due to their studies, experiences and researches. The snowball method was used to identify these experts. The inclusion criteria for the interviewees were having at least five years of work experience and knowledge on the sheltering of refugees due to conflict.

### Data collection

Following the purposive sampling of the participants, face-to-face interviews were conducted and recorded using an interview guide. Initially, two unstructured interviews were conducted to determine the main line of interview and complete the questions of the interview guide. Next, semi-structured interviews continued with 18 participants until data saturation when no new codes were detected and all study questions were answered.

At the beginning of the interviews, the participants were briefed on the objective of the study as to identify the criteria for the sheltering of refugees due to war or conflict on the assumption of entering Iran from the borderline. Then, the interviews continued with the following main questions: 1) which sites do you suggest for temporary accommodation? 2) Why do you suggest these sites? 3) Which are the characteristics of temporary sheltering of refugees in your view? They were also asked to give their views on socioeconomic, cultural, environmental, geographical, infrastructural, political, security, and health issues. The researchers asked the participants for clarifications on any ambiguous points during the interviews. At the end of the interviews, the participants were asked to introduce any other experts they knew on the subject.

The interviews were conducted from August to October 2020. Each interview lasted 25–60 min and 35 min on average. The interview audio files were listened to several times to confirm the accuracy of the data and the interviewer’s mastery, and the interviewees were contacted again in cases of ambiguity on important issues or new questions arising. All the interviews were recorded and transcribed verbatim. Data collection continued until data saturation.

## Data analysis

Data collection and analysis were conducted simultaneously in the present study using the Graneheim content analysis approach [18]. The analysis unit in the study was the recorded interviews which were transcribed verbatim, and repeatedly read and examined. The terminology, semantic units, and code extractions were carried out by the researcher and supervised by qualified professors in the field of qualitative study. Accordingly, the semantic units were identified as phrases or paragraphs in the transcripts and every key word or key phrase was coded. In the next step, similar primary codes were categorized to form primary categories. Then, similar subcategories were merged through constant comparative method of qualitative analysis to form the main categories. As each interview was added, constant analysis continued, and the codes and categories were modified. For a more accurate understanding of the concepts and to avoid superficial, mechanical coding, the coding and categorization process for the concepts were performed manually with paper and pencil.

### Trustworthiness

The trustworthiness of a qualitative research depends on the accuracy of the methodology [19]. Data were validated in the present research through four criteria of credibility, dependability, transferability, and confirmability.

### Credibility

To increase credibility, the researcher had a prolonged engagement from the initial stage of planning to data collection, qualitative analysis, and writing the manuscript. Member check was also used for rigor and trustworthiness. Our findings were presented to a number of participants to give their views on how the results projected their views on the temporary sheltering of refugees. Their views were used to modify the data accordingly. Peer debriefing was another method to increase data credibility. To this end, the colleagues in the research team examined the transcripts, codes and categories to correct any possible errors. The logic of repeated reviews, continuous comparison of data, summarizing and classifying information, and establishing a logical order among them without damaging the data determined the process of data validation.

### Dependability

Besides using the views of the research team, the outcome of data analysis was given to other qualitative researchers to obtain their complementary critique and ensure compatibility with the views of the research team.

### Transferability

To ensure transferability of the data, the following methods were used: simultaneous collection and analysis of data, ensuring coherence between the questions and methodology, comparing the results of the study with other studies, and reporting research stages step-by-step.

### Confirmability

The researcher's interest in the study phenomenon, their long-term engagement, transcribing the text of the interviews, and obtaining additional critical opinions by the participants, research team members and other qualitative researchers ensured the confirmability of this study.

## Results

The 20 participants were aged 33–70, with a median age of 48. Five of the participants were graduates, six were postgraduates, and nine had PhDs. From the analysis of the interviews, nine main categories, 53 subcategories, and 188 factors were extracted depicting the criteria for the sheltering of refugees due to conflict.

This study was compiled using interviews with experts, managers, and opinion holders of various state departments and organizations as described in Table 1.

The results of the interview analyses and complete description of the categories and subcategories, together with the factors is available in Table 2.

### Land characteristics

The first issue in sheltering refugees is the land on which they are to be resettled. The majority of the participants considered land type, slope, area, topography, height, and vegetation as the land criteria affecting the sheltering of refugees due to conflict.

One of the participants in the healthcare group stated: ‘In mountainous areas, if a population is accommodated on rocky land, it will be difficult to drain the sewage.’

Another important point mentioned in the land criteria was the proportionality of the land area to the displaced population. One of the participants stated: ‘Because we want to provide basic services, we must also consider the space per capita.’

### Access to infrastructural factor

Access to minimum infrastructures is needed to live in a refugee camp. The effective criteria for the sheltering of refugees due to conflict included in this study are water, electricity, gas, sewerage, roads, healthcare facilities, and means of communication.

One of the participants in the healthcare group stated: ‘Access to water, roads, and services must be available and the refugees must not encounter difficulties in this respect.’

Another participant in the accidents and disaster response group stated: ‘To create a site for shelter refugees, a small town must be constructed at the heart of another city with all its facilities, such as healthcare, clinics, water, gas, electricity, telephone, food, and clothing.’

### Access to facilities factors

Once the refugees have been accommodated, they need the least welfare facilities like the local population, especially for a long-term stay. The criteria for access to facilities for the sheltering of refugees in this study include recreation, shopping, religious-cultural, food, media, and firefighting. One of the participants in the relief and rescue group stated on the means of communication: ‘The problem of cell phone signals must be resolved because these people have to inform their kith and kin of their location’. Another participant talked about health: ‘we should not only pay attention to political and security issues. Rather, the required services, including welfare, health-treatment, counseling, water and nutrition services of the refugees should be taken into consideration’. Also, another participant says: ‘Temporary sheltering should be built at a suitable distance from the border. Because their entry into the country brings both health and security issues’.

### Homogeneity factors

Given the views of the participants, one of the important criteria for the sheltering of refugees due to conflict is homogeneity and similarities, including ethnicity, language, geography, religion, and culture. If these criteria are not observed in accommodating them, it may lead to difficulties in their resettlement or even acceptance. Homogeneity criteria affecting the sheltering of refugees due to conflict are sociocultural, religion, language, ethnicity, and geography. One of the participants stated on Iraqi refugees: ‘They made friends because they spoke Kurdish across the border. They felt comfortable together rather than strangers. Some of the families even said that they would not return to Iraq. Their common culture and language kept them together for years.’

Another participant in the relief and rescue group stated: ‘Usually, us Kurds on this side of the border, or the Baluchis on this side of the border, feel an affinity with those on the other side. Our society wants to accommodate them alongside us because race, ethnicity, and culture affect their resettlement.’ One of the participants says that ‘we should pay attention to which areas their social status, religion, and language are more inclined to. He also continues to choose their place of residence in such a way that they are compatible with each other, even their clothing and food’.

### Security factors

The safety and security of the refugees and that of the host country are urgent and important. According to the views of participants in the study, security factors affecting the sheltering of refugees include distance from sensitive areas, distance from the border, distance from the center of the country, control issues, international borders, and weapons manufacturing sites. One of the expert participants in the Aliens and Foreign Immigrants Affairs group stated: ‘It is best for the location of the site to be a day’s distance away from the border to keep a minimum level of safety for the displaced persons from any groups trying to find them. Travel must also be controlled and the camp site must be at a logical distance from the border.’

Another participant in the relief and rescue group stated on the existing refugees in Iran: ‘A huge safety concern for Afghan refugees created in Iran later on was that permission was given for Afghan and Iraqi refugees to move far away from the border areas and resettle in other cities. They still have many problems even today. Refugees must not be moved too far away from the border areas and into the heart of the country.’

### Hazard safety factors

To avoid the manifold pain and suffering of refugees, they must be kept safe from subsequent dangers. According to the views of the participants, the criteria affecting distance from danger for the accommodation of displaced persons include floods, volcanic eruptions, wind currents, avalanches, landslides, faults, dams, noise, high voltage power lines, radioactive material, mines, area of diseases and carriers, and wild animals. One of the participants in the healthcare group stated: ‘The camp site must not be exposed to floods or endemic diseases such as cutaneous leishmaniasis.’

Another participant in the accidents and disaster response group stated: ‘We all think about earthquakes. We must investigate that these resettlement sites are not located on faults, or at least not on active faults.’

### Environmental factors

Environmental criteria are important factors which must be considered when accommodating refugees. According to the participants, environmental factors affecting the sheltering of refugees include protected areas, rare plants, and local wildlife. One of the participants in the healthcare group stated: ‘Environmental factors are important when accommodating displaced persons. Presently, environmental organizations in all countries have their own particular rules and regulations. We may not be aware that a place is a protected area in one country and accommodate refugees in that area because we think it is a suitable plain with water. But we are not aware that this is a protected area.’

### Economic factors

The economic factors in a host country and the livelihood of the displaced persons in the camps must not be neglected. According to the participants, economic criteria affecting the sheltering of refugees include costs, economic efficiency, and job prospects. One of the participants in the Aliens and Foreign Immigrants Affairs group stated: ‘The upkeep of camps in costly. When the refugees leave the camps, we can rent these out to newlyweds for a nominal rent to cover the lateral costs of the camp. This will help people and avoid the place from becoming derelict at the same time.’

### Political and legal factors

It is important to observe the rights of the landowner where the refugees are accommodated to prevent possible tensions afterwards. According to the participants, the legal criteria for land ownership affecting the sheltering of refugees are state, public, and privately owned land. One of the participants in the healthcare group stated: ‘The land allocated to sheltering refugees must not have legal issues. On numerous occasions, refugees have been accommodated and tents set up in a farmer’s agricultural land. This creates local disputes and complaints by the farmer. If a farmer’s land is to be used, the legal issues must be cleared up and consent received.’

## Discussion

Given the growing number of refugees due to conflict and war in the world, this study was conducted in Iran to identify the criteria for the sheltering of refugees due to conflict, using the views of experts and opinion holders. A wide range of criteria for the temporary sheltering of refugees due to conflict was extracted and categorized in the present study. Extracting the criteria using the opinions of different people from most organizations involved in the sheltering of refugees due to conflict is one of the strengths of this qualitative study. Many of these criteria are consistent with the criteria mentioned in international reliable sources such as UNHCR, but in addition to the common criteria, this study has stated other important necessary criteria (Table 3). This study has addressed the necessary and important details in the temporary sheltering. Qualitative studies are suitable for creating a comprehensive understanding of the researched phenomenon based on the experiences and knowledge of the participants in the context of the society [16]. Other studies conducted such as the studies of Jana Abikova, 2020 [13] and Çetinkaya et al., 2016 [15] are descriptive and most of them have been done by distributing questionnaire, in which people don’t have the possibility to express their personal opinions and only have the possibility to response on the questions that made by the researchers. Whereas, in this qualitative study, a wide range of people from different organizations participated and interviewed. As Table 3 shows, the criteria obtained from this study are more comprehensive and complementary to other studies.

**Table 1:** Number of sessions with the study subjects according to the city and study group

**Study Group**												
City	Experience in Sheltering		The Expertise of the participants									
	Yes	No	International Relations	Agriculture	Electricity	Water	Accidents and disasters	Political and security	Immigration Affairs	Relief and Rescue	Healthcare	**Total**
Tehran	2	2	-	-	-	-	1	-	-	3	-	**4**
Sanandaj	4	2	1	-	-	-	1	1	1	2	-	**6**
Ghorve	1	1	-	-	-	-	1	-	-	-	1	**2**
Dehgolan	5	-	-	1	1	1	-	1	-	1		**5**
Marivan	-	1	-	-	-	-	-	-	-	-	1	**1**
Kamyaran	-	2	-	-	-	-	-	-	–	-	2	**2**
Total(%)	12(55%)	8(45%)	1(5%)	1(5%)	1(5%)	1(5%)	3(15%)	2(10%)	1(5%)	6(30%)	4(20%)	**20(100%)**

**Table 2:** Categories, subcategories, and codes for the refugees sheltering

Category	**Sub-Category**	**code**
**Land characteristics**	Soil texture	The rockiness of the land, The clay of the land, The barrenness of the land, Residential capability of the land, Swampy land
	Land slope	Suitable land slope
	Land area	Sufficient area, Proportion of area to population (population density)
	Earth topography	Considering the lowness and highness of the ground, land use, Considering the land forms
	Elevation	Having a suitable height, located at appropriate height, The ground should not be too flat
	Vegetation cover of ground	Sufficient vegetation cover, Not located in the forest area
	Location of land	Lack of conflict in the area, Specific location of the camp, Sufficient light, Considering all the needed facilities for a city
**Access to infrastructural factor**	Water	Possibility of water supply, Having water resources, Possibility of easy water supply, Access to piping, Access to healthy water,
	Electricity	Availability of electricity supply, Access to electricity, Existence of power transmission lines, Proximity to power supply
	Sewage disposal	Availability of sewage disposal system, Not exposed to sewage, Existence of sanitary services
	Trash disposal	Access to the garbage collection system, Having a place for landfilling, Possibility of landfill
	Road	Access to the main road, Short distance to the road, Safe distance from the road, Standard distance from the road, Access to transportation facilities, having transportation routes
	Gas	Access to gas, Possibility of gas supply, Suitable distance from gas transmission lines
	Health facilities	Having health facilities, Access to medical services, Access to hospital, Access to pre-hospital emergency, Setting up a health system, Providing welfare and health services, Having an ambulance, Having a field hospital, Access to medicine
	Communication equipment	Telephone, Internet, Telecommunications, Mass media, Electronic communications, Communication devices
**Access to facilities factors**	Welfare – recreation facilities	Stadium, bathroom, Recreational facilities, Places for children to play, Park, Sports field, Storage, Housing and shelter, Tent, Residential building, Canopy,
	Shopping facilities	Existence of local shopping centers, Distance from shopping malls, Access to shopping centers
	Religious-cultural place	Access to a mosque or place of worship, Existence of a hall for religious meetings and rituals, Existence of a place for burial of corpses, Possibility of burial of corpses
	Food Supply Facilities	Existence of food storage, Access to food storage, Access to food, Existence of food source
	Media Facilities	TV accessibility, Radio accessibility
	Firefighting Facilities	Proximity to fire stations, Fire safety
	Access Status	Proximity to the city, Proximity to the village,
**Homogeneity factors**	Sociocultural Factors	Cultural adaptation, Hospitality of the host, Closeness of the culture, Acceptability of the culture, Level of literacy, Culture within the IDPs, Satisfaction of the host community, Recognition of social status, Attention to identity, Consideration of individuals, Social support, Notoriety of society, Demographic composition
	Religious Factors	Considering religious prejudices, Absence of religious conflict, Considering beliefs
	Language	Linguistic similarity, Co-language, Linguistic commonalities
	Ethnicity	Ethnic commonalities, Absence of ethnic conflicts
	Geographic similarity	Similarity of geographic, climate and weather with the origin of the refugees
**Security factors**	Distance from critical structures	Distance from the cemetery, Distance from the city's drinking water source, Distance from military centers, Distance from sensitive factories, Distance from historical places, Distance from religious centers
	Distance from the border	Appropriate distance from the border, located in border provinces while maintain distance from the border (Not located in central provinces),
	Distance from the center of the country	Distance from the center of the country
	Safety	Considering security issues, Controlling entry and exit, having a fence, Protection around the camp, Controlling traffic, Controlling the length of stay of refugees
	Host Country	Not war-torn country
	Existence of War Weapons	Mined neighborhoods, Barbed wire lands
**Hazard safety factors**	Flood	Not locating in flood-prone areas, Not being in the flood path, Not being in the river path, Not being in the alluvial path, Not being in the river bank, Not being in the vicinity of a river
	Volcano	Not exposed to volcanoes
	Storm	Not exposed to storms
	Mountain Landslide	Not exposed to mountain collapse and rock fall
	Landslide	Not exposed to landslide risk
	Faults	Not prone to seismicity risk, Not being on a fault, Distance from fault
	Dam	Not located in downstream of the dams, Not located near or adjacent to the dam
	Power Lines	Distance from high voltage electricity, Not located under high voltage electricity, Distance from power line
	Radioactive Activities	Not exposed to radioactive radiation, Not be at risk of nuclear explosion
	Mines	Distance from mines
	Environmental Pollutions	Distance from sound source, Distance from noise pollution, Distance from air pollution
	Diseases	Considering endemic diseases of the region, Possibility of preventing diseases, Considering contagious diseases, Considering insect vectors, Considering vermin
**Environmental factors**	Status of Environmental Protection	Environmentally protected area
	Considering the Rare Plants	Considering the rare plants, Protection of rare plants
	Considering the Wildlife	Considering rare animals, Protection of environmental, Considering carnivores, Considering rodents, wild animals
	Climate of the Region	Good weather of region, Clean air
**Economic factors**	The Economic Situation	Economic efficiency, The versatility of the Camp, Host economic situation, Access cost
	Possibility of Job Activity	Access to employment،Availability of income-generating activities, Safe and healthy working conditions, Access to local factories
**Political and legal factors**	Legal Status of The Land	Governmental possession, National possession, Public possession, Private sector possession
	Political Issues	Political policy of hosting country, Having refugees Action plan, Deciding on the location of the camp by local authorities

**Table 3:** The comparison of the criteria obtained from this study with UNHCR criteria

Criteria	UNHCR	This study	Explanation
Soil Texture	Yes	Yes	Explained in general in UNHCR, in this study given as detail
Land Slope	Yes	Yes	In this study, the suitability of the land slope is mentioned, but UNHCR has revealed in more detail the slope of the land and its degree in different situations
Land Area	Yes	Yes	In this study, the proportionality of the land area with the population (Population density) is emphasized
Earth Topography	Yes	Yes	In this study, topography is defined as land use, land forms and lowness and highness of the ground
Vegetation of the Earth	Yes	Yes	In this study, the necessity of sufficient vegetation cover is mentioned, it is also stated that shelters should not be located in areas with disturbing vegetation such as forest areas
Elevation	Yes	Yes	In this study, the meaning of elevation is explained: the land of the shelter should not be flat and should have a suitable height
Access to water	Yes	Yes	-
Access to Electricity	Yes	Yes	In this study, different forms of access to electricity are mentioned: Access to electricity supply, power transmission lines, and power supply
Drainage	Yes	Yes	In this study, the type of land and availability of sewage disposal system are given
Access to Road	Yes	Yes	In this study, access to the road is considered as one of the essential infrastructures Moreover, road features for temporary shelter are explained
Access Health Facilities	Yes	Yes	In UNHCR, proximity to health services is mentioned in general as a complementary or supportive factor, but in this study, access to health facilities is mentioned in details. In addition, in this study, access to health facilities is mentioned as one of the necessary infrastructures
Education services	Yes	No	Although the provision of educational services is not mentioned in this study, access to schools and educational facilities is definitely one of the important criteria in temporary sheltering
Availability of other UN agencies NGOs implementing partners, operating partners and humanitarian groups,	Yes	No	Although this criterion is not mentioned in this study, according to UNHCR, it is considered a supportive factor in temporary sheltering
Firefighting Facilities	Yes	Yes	Both this study and the UNHCR have mentioned the fire safety, in UNHCR more details are mentioned such as considers fire risk mitigation strategies, distances of firebreak In this study, the delivery of fire services is considered as the proximity to fire stations, this criterion is in the category of access to public services
Proximity to The City	Yes	Yes	In UNHCR, it is mentioned as a support factor, but in this study, it is mentioned in the category of access to public services
Proximity to the Village	Yes	Yes	In UNHCR, it is mentioned as a support factor, but in this study, it is mentioned in the category of access to public services
Sociocultural Factors	Yes	Yes	In this study given as detail
Distance from Important Centers	Yes	Yes	In this study given as detail
Distance from the Border	Yes	Yes	In UNHCR, the distance from international borders and sensitive sites are mentioned together, but in this study, these two criteria are explained separately and in more detail
Mines	Yes	Yes	In this study, distance from mines is mentioned only. But the UNHCR has described it in more detail, such as distance from landmines and unexploded ordnance
Status of Environmental Protection	Yes	Yes	UNHCR mentioned it in general but this study examines it in detail
Climate of the Region	Yes	Yes	UNHCR mentioned it in general but this study examines it in detail
Possibility of job activity	Yes	Yes	In the UNHCR the proximity to the economic centers mentioned to make money
Legal status of the land	Yes	Yes	In this study, the details of land ownership are mentioned
Security factors	Yes	Yes	It is the same for both, but explained in general in UNHCR, in this study given as detail
Considering endemic diseases of the region	Yes	Yes	In UNHCR, the examination of the local health and other risks is mentioned in general, but this study has given special attention to endemic diseases
Harvesting wood for construction	Yes	Yes	In this study, other fuel sources are mentioned, including gas
Harvesting wood as cooking fuel	Yes	Yes	In this study, other fuel sources are mentioned, including gas
Length of stay of refugees	Yes	Yes	In UNHCR, the length of stay is considered as one of the factors related to potential beneficiaries. But in this study, this criterion is mentioned in the category of security and as one of the control issues
Population	Yes	Yes	The number of populations is one of the effective factors for the size of the land
The type or category of people we are planning to assist	Yes	Yes	In this study in the sociocultural factors mentioned
Access to gas	Yes	Yes	In the UNHCR, other fuel sources are mentioned, including wood for the cooking and construction
Distance to trash disposal (garbage)	NO	Yes	Not mentioned in UNHCR
Access communication equipment (Telephone, Internet)	Yes	Yes	UNHCR has explained in more detail
Welfare – Recreation Facilities (Stadium, Shopping centers …)	Yes	Yes	Although UNHCR has emphasized on the welfare of refugees, this study mentions the provision of more facilities such as stadiums and shopping centers
Religious-Cultural place	NO	Yes	Although UNHCR has paid attention to the religious and cultural issues, it has not mentioned the provision of a place to carry out religious and cultural activities
Food Supply Facilities	NO	Yes	It is not mentioned directly in UNHCR
Media Facilities	Yes	Yes	In addition to access to the media, UNHCR has mentioned access to new international media as a source of information
Homogeneity Religious Factor	NO	Yes	Although the basic principles of refugees' religious activities have been raised in UNHCR and their rights have been defended, homogeneity religious factor has not been mentioned. In fact, this criterion can be considered as a supportive factor
Homogeneity Geographical	Yes	Yes	UNHCR has addressed the issue of language in refugees in more detail
Homogeneity Ethnicity	NO	Yes	Although UNHCR has paid attention to the issue of ethnicity, it has not mentioned the homogeneity of the refugees' ethnicity with the host country. This criterion can be considered as a supportive factor
Homogeneity Language	Yes	Yes	UNHCR has paid attention to the issue of language in sheltering in more detail, and in addition to linguistic commonalities, it has mentioned the rights of refugees, communication barriers related to language and the language threats of the host country
Host Country is not War-Torn Country	NO	Yes	UNHCR has mentioned the security of the region in general
distance Volcano	NO	Yes	In the UNHCR the distance from natural hazards is generally mentioned
distance Storm	NO	Yes	
distance Faults	NO	Yes	
Mountain Landslide	NO	Yes	
Flood	Yes	Yes	-
Landslide	Yes	Yes	-
distance Dam	NO	Yes	It is not mentioned directly in UNHCR
Considering the rare plants	NO	Yes	It is not mentioned in UNHCR
Considering the Wildlife	NO	Yes	It is not mentioned in UNHCR
distance Power Lines	NO	Yes	It is not mentioned directly in UNHCR
Distance Radioactive Activities	NO	Yes	It is not mentioned directly in UNHCR
Political Issues	NO	Yes	It is not mentioned directly in UNHCR
Environmental Pollutions	Yes	Yes	In UNHCR, it has mentioned the shelter's protection against environmental pollution, especially water resources. But this study has pointed out to stay away from other environmental pollutions such as noise pollution and air pollution

Various studies show that the land allocated to sheltering needs due attention in accommodating refugees [1, 5, 13, 14, 20–24]. According to our study, the most important properties of the land selected for refugee camps are land type, slope, area, topography, height, and vegetation. The results of our study show that rocky land, prairies, and forests are not suitable sites. By the same token, arable land, and land with steep slopes or without slopes are also not suitable sites for camps.

Security is one of the factors which must be given serious consideration when sheltering refugees due to conflict. The concerns of refugees about the security issues are controversial because security may prevent them from returning to their community [1, 5, 23]. As they have been displaced due to manmade conflicts, the host State and local population must guarantee their safety and security. Criteria such as distance from the conflict areas in the study conducted by Çetinkaya et al. [15], and a logical distance from the border in the study by Drakaki et al. [14] have been reported. Based on the results of the present study, host countries and communities must not guide and accommodate refugees in the center of the country and its community. Our results show that displaced persons due to conflict must be accommodated at a logical distance from the border, away from the firing range of the conflict area to avoid being chased by hostile groups. It is possible for saboteurs to create insecurity in the host country disguised as displaced persons. Therefore, the entrance and exit of displaced persons must be under control and the accommodation area must be delineated. Moreover, according to the results of this study, the site of the accommodation must be at a logical distance from important centers for the sustained security of the host community. These include the drinking water facilities of a city, garrisons, security centers, and historical, cultural, and religious sites.

Homogeneities and similarities in this study include uniformity, homogeneity, resemblances, and consistencies among displaced persons and the host community. These criteria must be taken into consideration when sheltering refugees due to conflict. A shared culture, history, and religion in accommodating refugees have been mentioned in some studies and documents [5, 24, 25]. The results of this study show that a common language, ethnicity, and sociocultural traditions between the refugees and the host community bring acceptance and a longer stay. Shared similarities mean that a number of refugees may stay in the host community forever and become part of it. In addition to compatibility, the said similarities also means mutual benefits of employment and income for the refugees, as well as the host community benefiting from the skills of the refugees. For instance, it might be better to accommodate Kurdish-speaking refugees in Kurdish speaking areas, Baluchi-speaking persons in Baluchi speaking areas, and Arabic-speaking persons in Arabic speaking areas, where they share more similarities with each other. Geographical similarity is also another issue which must be considered. In a study by Nappi and Souza [12], the suitability of the site sheltering refugees must match their climatic conditions. In other words, persons displaced from tropical regions must not be accommodated in cold regions and vice versa, because this will increase their pain and suffering.

The hazard safety factor for sheltering refugees due to conflict were mentioned by most participants in the study. Staying away from natural hazard prone areas and any real and potential dangers have also been mentioned in other studies and documents [4, 5, 7, 9, 10, 15, 26–28] as criteria for accommodating refugees. The findings of the present study also show that apart from a logical distance from the conflict and warring areas, such as mine fields, the location must also be a safe distance from natural hazards, such as floods, faults, volcanos, avalanches, cliffs, landslides, dams, mines, inflammable and radioactive materials, and noise pollution.

Some studies have mentioned the distance between the site sheltering refugees with high-risk areas for diseases such as malaria [5, 14]. The results of this study also show that a safe distance must be kept between the site sheltering refugees and high-risk areas for native diseases such as cutaneous leishmaniasis and malaria. Currently, observing COVID-19 protocols for the health of refugees and the host community is also vital. Refugees must also not be accommodated in areas where there is a danger of attack by wild animals.

Access to infrastructures and welfare facilities is another factor considered in certain studies on sheltering refugees [4, 5, 10, 11, 14, 23, 26, 28–30]. Furthermore, our results show that the infrastructures required for sheltering refugees due to conflict must be provided like a city on a smaller scale. These include water, gas, electricity, telephone, internet, school, sports center, park, playground, silos, buildings, and tents. Of course, it is noteworthy that should the facilities provided for the shelter of refugees due to conflict be more than what is available to the local community, this may lead to dissatisfaction in native residents and even create conflict with the local authorities.

It is necessary to consider environmental factors in sheltering refugees due to conflict [5, 11, 12, 22]. According to the results of this study, sheltering refugees must not damage and destroy the rare fauna and flora of the region. The location must also not be a protected area. Some studies have also indicated that the refugees must take measures to protect the environment and prevent pollution [14, 25]. This is also compatible with our study.

Decision-making in selecting a site for the shelter of refugees is outside the scope of a single organization. Rather, it is a team work which must be accomplished using the knowledge of various disciplines in coordination with all the organizations involved. A study by Liu et al. [7] on minimum humanitarian standards also shows that the knowledge of other disciplines must be used, and this is also compatible with the findings of the present research. In fact, the results of this study show that sheltering refugees due to conflict with a view to creating a city for a long-term stay must take place by a range of service companies and experts in resettlement.

The main organizations involved in this research were the Water and Wastewater Company, Power Generation and Distribution Company, Ministry of Roads and Urban Development, Telecommunication Company, Ministry of Agricultural Jihad, crisis management and defense, Bureau for Aliens and Foreign Immigrants Affairs, political and security authorities, and experts of accidents, disasters, and natural disasters response.

The main limitations of the present study were at the time of the interviews, the refugees were not in the camps of the study area. In addition, it was not possible to access the former refugees in the neighboring countries due to the conditions of the Covid-19 pandemic.

## Conclusion

A range of factors must be considered when sheltering refugees due to conflict. It is necessary for policymakers, managers, and decision-makers in the country to take these criteria into consideration when planning to shelter refugees. Moreover, it is necessary to create the economic and functional infrastructures to enable Iran to accept refugees. The results of this study indicate that improving the process of sheltering refugees due to conflict requires extensive intraorganizational and interorganizational cooperation and orchestration across the country.

Therefore, it is suggested that the essential training, guidelines, planning, and resources be provided and implemented.

## Data Availability

The datasets used and/or analysed during the current study are available from the corresponding author on reasonable request.
